# Influence of rosuvastatin treatment on cerebral inflammation and nitro-oxidative stress in experimental lung injury in pigs

**DOI:** 10.1186/s12871-021-01436-0

**Published:** 2021-09-13

**Authors:** Jens Kamuf, Andreas Garcia Bardon, Alexander Ziebart, Robert Ruemmler, Johannes Schwab, Mobin Dib, Andreas Daiber, Serge C. Thal, Erik K. Hartmann

**Affiliations:** 1 Department of Anesthesiology, University Medical Centre, Mainz, Germany; 2 Department of Cardiology, University Medical Centre, Mainz, Germany

**Keywords:** Acute respiratory distress syndrome, Pigs, Rosuvastatin, Nitro-oxidative stress, Inflammation

## Abstract

**Background:**

Many patients with acute respiratory distress syndrome (ARDS) suffer from cognitive impairment after hospital discharge. Different mechanisms have been implicated as potential causes for this impairment, inter alia cerebral inflammation. A class of drugs with antioxidant and anti-inflammatory properties are β-HMG-CoA-reductase inhibitors (“statins”). We hypothesized that treatment with rosuvastatin attenuates cerebral cytokine mRNA expression and nitro-oxidative stress in an animal model of acute lung injury.

**Methods:**

After approval of the institutional and state animal care committee, we performed this prospective randomized controlled animal study in accordance with the international guidelines for the care and use of laboratory animals. Thirty-two healthy male pigs were randomized to one of four groups: lung injury by central venous injection of oleic acid (n = 8), statin treatment before and directly after lung injury (n = 8), statin treatment after lung injury (n = 8), or ventilation-only controls (n = 8). About 18 h after lung injury and standardized treatment, the animals were euthanised, and the brains and lungs were collected for further examinations. We determined histologic lung injury and cerebral and pulmonal cytokine and 3-nitrotyrosine production.

**Results:**

We found a significant increase in hippocampal IL-6 mRNA after lung injury (p < 0.05). Treatment with rosuvastatin before and after induction of lung injury led to a significant reduction of hippocampal IL-6 mRNA (p < 0.05). Cerebral 3-nitrotyrosine was significantly higher in lung-injured animals compared with all other groups (p < 0.05 vs. animals treated with rosuvastatin after lung injury induction; p < 0.001 vs. all other groups). 3-Nitrotyrosine was also increased in the lungs of the lung-injured pigs compared to all other groups (p < 0.05 each).

**Conclusions:**

Our findings highlight cerebral cytokine production and nitro-oxidative stress within the first day after induction of lung injury. The treatment with rosuvastatin reduced IL-6 mRNA and 3-nitrotyrosine concentration in the brains of the animals. In earlier trials, statin treatment did not reduce mortality in ARDS patients but seemed to improve quality of life in ARDS survivors. Whether this is attributable to better cognitive function because of reduced nitro-oxidative stress and inflammation remains to be elucidated.

**Supplementary Information:**

The online version contains supplementary material available at 10.1186/s12871-021-01436-0.

## Background

The acute respiratory distress syndrome (ARDS) is a complex disease pattern with multi factorial origin. It is defined by the Berlin definition according to clinical features, namely acute onset of respiratory failure, not fully explained by cardiac failure, and bilateral opacities in thoracal imaging, not fully explained by effusions, atelectasis, or nodules [1]. Pathological there are three key components: 1. Pulmonal inflammation indicated by a neutrophilic alveolitis, 2. Disruption of the alveolar-capillary barrier with deposition of hyaline membranes in the airspace, 3. Endothelial injury with activation of the coagulation cascade and formation of microthrombi [2]. Endothelial damage can lead to oxidative stress which aggravates inflammation, endothelial damage and the severity of the acute lung injury [3].

Patients with acute respiratory distress syndrome not only need intensive care and mechanical ventilation but also very often suffer from cognitive impairment after hospital discharge [4, 5]. The cause for this cognitive impairment is still not known, but several mechanisms, such as hypoxia [4], inflammation [6], or nitro-oxidative stress, are under discussion [7]. Another possible cause for cognitive impairment in ARDS could be mechanical ventilation itself, as this was shown to induce hippocampal apoptosis by vagal pathways [8], and hippocampal damage is associated with cognitive decline [9, 10]. Furthermore, the hippocampus is thought to be especially susceptible to hypoxia [11]; thus, hypoxia and mechanical ventilation should be even more deleterious for the hippocampus. Bickenbach et al. showed in a pig study that there is a difference in the results of neurologic tests after hypoxia due to a reduced fraction of inspired oxygen (FiO_2_) and hypoxia due to HCl-induced lung injury [12]. They did not find a difference in hippocampal damage, even though the animals were ventilated with rather high tidal volumes (10 ml/kg) and without positive end-expiratory pressure (PEEP), which in itself should induce hippocampal damage, according to the aforementioned results.

β-HMG-CoA-reductase inhibitors (statins) are a class of drugs with pleiotropic effects. They reduce serum cholesterol and also show anti-inflammatory and antioxidant properties. There is evidence of neuroprotective effects of statins [13]. Some studies have demonstrated promising results for sepsis- and ventilator-induced ARDS [14, 15], suggesting that these drugs could be beneficial to patients with ARDS [16, 17]. However, randomized controlled trials (RCTs) showed no significant differences regarding mortality, ventilator-free days, or length of hospital stay [18, 19]. A retrospective analysis of these studies after screening for hypo- and hyperinflammatory subgroups showed a significant reduction in patients with hyperinflammatory ARDS treated with simvastatin, but no effect in patients with sepsis-induced hyperinflammatory ARDS treated with rosuvastatin [20]. One retrospective study investigating the effect of statin therapy on cognitive impairment after sepsis-induced ARDS did not show convincing results [21]. Other studies have shown an improvement in the quality of life in simvastatin-treated patients with ARDS [22]. Nevertheless, the role of statins in ARDS remains controversial [23–28].

Thus, there are many uncertainties about the concurrent cognitive decline in ARDS and the potential benefit of statins—not in terms of reducing mortality or time to clinical discharge but after returning home—for patients with ARDS. We hypothesised that cerebral cytokine production and nitro-oxidative stress could be attenuated by early treatment with rosuvastatin.

## Methods

After approval by the institutional and state animal care committee (Landesuntersuchungsamt Rheinland-Pfalz, Koblenz, Germany; approval number G15-1–077), we performed this prospective randomized controlled animal study in accordance with the international guidelines for the care and use of laboratory animals and in compliance with the ARRIVE guidelines. This manuscript adheres to the applicable EQUATOR guidelines. Some of the animals used in this study were also part of other studies [29].

### Anaesthesia and instrumentation

Thirty-two healthy male pigs (sus scrofa domestica, weight: 26–33 kg) were randomized to one of four groups: lung injury by central venous injection of oleic acid (OAI, n = 8), statin treatment 12 h before and directly after lung injury (SBA, n = 8), statin treatment after lung injury (SA, n = 8), or ventilation only (VO, n = 8).

The whole study and post-mortem analyses were done investigator-blinded. The person responsible for the treatment with rosuvastatin was not involved in any other part of the study.

Anaesthesia and instrumentation were conducted as described before [30]. All animals were delivered sedated (4 mg kg^−1^ ketamine, 8 mg kg^−1^ azaperon intramuscular) by a local breeder. After establishing an intravenous line, anaesthesia was induced and maintained by propofol (8–12 mg kg^−1^ h^−1^) and fentanyl (0.1–0.2 mg kg^−1^ h^−1^). A single dose of atracurium (0.5 mg kg^−1^) was administered to facilitate orotracheal intubation. Ventilation (respirator: Engström Carestation®, GE Healthcare, Germany) was started in pressure-controlled mode with a tidal volume (V_t_) of 7 ml kg^−1^, PEEP of 5 cmH_2_O, FiO_2_ of 0.4, and a variable respiratory rate to maintain normocapnia. A balanced electrolyte solution (Sterofundin ISO, B. Braun, Germany) was continuously infused at a rate of 5 ml kg^−1^ h^−1^. Vascular catheters were placed ultrasound-guided: an arterial line, a pulse contour cardiac output catheter (PiCCO, Pulsion Medical Systems, Germany), a central venous line, and a 7.5 French introducer for a pulmonary arterial catheter were inserted via femoral vascular access. Respiratory and extended hemodynamic parameters were recorded continuously (Datex S/5, GE Healthcare, Germany). The PiCCO was calibrated regularly for measurement of extravascular lung water index (EVLWI) as marker for pulmonary oedema and cardiac index (CI) to rule out acute cardiac failure. Further respiratory parameters and measurements were recorded by the respirator. Normothermia was maintained by body surface warming.

### Study protocol

The study protocol was described before [30]. Following instrumentation, we set the F_i_O_2_ to 1.0 and conducted a lung recruitment manoeuvre (plateau pressure 40 cmH_2_O for 10 s). Baseline parameters were then assessed at healthy state. For the animals in the lung injury and treatment groups (OAI, SBA, SA), 0.1 ml kg^−1^ of oleic acid (cis-9-octadecenoic acid) was dissolved in 20 ml saline solution and injected via the central venous line in fractions of 2 ml every 3 min. The procedure was repeated with another 0.1 ml kg^−1^ after 15 min if the PaO_2_/FiO_2_ was higher than 200 mmHg. After the ARDS criteria [1] were fulfilled, the animals were treated according to a standard protocol that was closely adapted to that used in human treatment in ICUs. The respirator was set at *V*_t_ 6 ml kg^−1^, FiO_2_ and PEEP, as displayed in Table 1, with an intended SpO_2_ of 94–98%. If necessary to warrant stable hemodynamics during the experiments (mean arterial pressure > 65 mmHg), norepinephrine was administered. We collected blood samples directly after induction of lung injury and 6, 12, and 18 h later. After 18 h, the animals were sacrificed under deep general anaesthesia by injection of 200 mg propofol and 40 mmol potassium.

**Table 1:** PEEP/FiO2 setting according to the low PEEP/high FiO2 table of the ARDSnet

FiO_2_	0.4	0.4	0.5	0.5	0.6	0.7	0.7	0.7
PEEP	5	8	8	10	10	10	12	14
FiO_2_	0.8	0.9	0.9	0.9	1.0	1.0	1.0	1.0
PEEP	14	14	16	18	18	20	22	24

### Rosuvastatin treatment

Animals of two groups were treated with rosuvastatin: the animals of the SA group and the animals of the SBA group.

The animals of the SA group received 1 mg/kg bodyweight rosuvastatin intravenously after induction of the lung injury. Rosuvastatin for intravenous treatment was dissolved in saline as described by Prinz et al. [31].

The animals of the SBA group received rosuvastatin at two different time points during the study. 12 h before the lung injury was induced, the animals were fed with one tablet rosuvastatin (25 mg). Additionally, the animals of this group received 1 mg/kg bodyweight rosuvastatin intravenously after induction of the lung injury. Rosuvastatin for intravenous treatment was dissolved in saline as described by Prinz et al. [31].

### Post-Mortem analysis

After death, the brains and lungs were harvested for further investigations. We obtained a defined slice of the frontal cortex and the hippocampus of the brain samples. All samples were shock frosted (in liquid nitrogen) for molecular biological analysis and assessment of nitro-oxidative stress.

The lungs were used for wet/dry ratio, histopathology, TNFalpha mRNA and nitro-oxidative stress measurements.

### Wet-to-dry ratio

The left lung was weighted immediately after removal and dried afterwards at 60 °C for 72 h to determine the dry weight and wet-to-dry ratio.

### Lung histopathology

After extraction of the lungs, dependent and non-dependent lung regions were sampled, fixed in formalin for paraffin sectioning and stained with HE. The lung injury score was assessed as previously described [32]. In short, 7 different parameters in 4 non-overlapping regions are evaluated by a blinded investigator and scored with a severity grade between 0 and 5 points. These points add up to the total lung injury score with a maximum of 140 points.

### Cerebral cytokine expression

To determine the cerebral production of mRNA of TNFalpha, IL-6, IL-8, and iNOS we used real-time polymerase chain reaction (RT-PCR, Lightcycler 480 PCR System, Roche Applied Science, Germany) as previously described [33]. mRNA expression data were normalized against peptidylprolyl isomerase A as a control gene. The sequences of the applied oligonucleotide primer pairs (5’-3’) are displayed in Table 2.

**Table 2:** Primer sequences for PCR

PCR Assay	Oligonucleotide Sequence (5 ‘-3 ‘)	Gene bank number
*PPIA*	fw-CTTTCACAgAATAATTCCAggATT	NM_214353
	rev-ggACAAgATgCCAggACC	
	fl-ATgCTTCAggATAAAATTCTCATCATCAAA	
	cy5-TTCTCTCCATAgATggACTTgCCACCA	
*IL 6*	fw-CCAATCTgggTTCAATCAggA	NM_214399
	rev-gTggTggCTTTgTCTggATTC	
	fl-TgTCgAggCTgTgCAgATTAgTACCA	
	cy5-gCACTgATCCAgACCCTgAggCAA	
*TNFalpha*	fw-CCCAgAAggAAgAgTTTCCA	NM_214022
	rev-CggCTTTgACATTggCTACA	
	fl-ggCCCAAggACTCAgATCATCgTC	
	cy5-CAAACCTCAgATAAgCCCgTCgC	
*iNOS*	fw-gATggCACCATCATAggggAC	NM_001143690
	rev-ggCACCCTgggAACTCAA	
	fl-TGGAACACCCCAAATACGAGTGGTTCC	
	cy5-GGAGCTGGAGCTGAAGTGGTACGCCC	
*IL8*	fw-CAAgAgTAAgTgCAgAACTTCgAT	NM_213867
	rev-CAggCAgACCTCTTTTCCAT	
	fl-CACCTTTCCACCCCAAATTTATCAAg	
	cy5-AACTgAgAgTgATTgAgAgTggACCCC	

### Serum cytokines

After ARDS induction, 6, 12, and 18 h later, blood samples were collected and snap frozen for determination of TNFalpha levels using ELISA kits (Porcine TNFalpha Quantikine ELISA, R&D Systems, Wiesbaden, Germany) according to the instructions of the manufacturer and earlier description [30].

### Nitro-oxidative stress

Nitro-oxidative stress was measured in lung and brain samples. Analysis of total protein homogenates was performed by dot blot as previously described [34]. For this, equal amounts of protein homogenates were diluted in phosphate-buffered saline and each sample applied to a buffer-soaked nitrocellulose membrane Protran BA85 (0.45 μm) using a Minifold I vacuum dot-blot system device (Whatman Schleicher& Schuell, Dassel, Germany) with a 96-well top frame. Each slot was washed with 250 μl PBS and the membrane was dried for 60 min at 60 °C. For detection of nitrated protein, a mouse monoclonal 3NT antibody (Millipore) was used at a dilution of 1:1000. Signals were detected by enhanced chemiluminescence after incubation with a peroxidase-coupled anti-mouse secondary antibody at a dilution of 1:5000 (Vector Lab, USA). Densitometric quantification was performed with a ChemiLux Imager (CsX-1400 M, Intas, Göttingen, Germany) and Gel-Pro Analyzer software (Media Cybernetics, Bethesda, MD).

### Statistics

The results were analysed by one-way ANOVA with post hoc tests for multiple testing (SNK), a p < 0.05 was considered significant. Sigmaplot 12.5 was used for analysation of the data and graphing the plots.

## Results

Oxygenation ratio dropped significantly and peak pressure increased significantly after induction of lung injury at all measured time points in OAI animals compared to ventilation-only animals. This effect was not affected by treatment with statins. Furthermore, induction of lung injury led to a significant increase in PEEP, fiO_2_, and EVLWI at certain time points compared to VO without amelioration by statin treatment. EVLWI was significantly higher in SBA compared to OAI at 0 h, 12 h and 18 h. Tidal volume, end-expiratory CO_2_, and wet-to-dry ratio didn’t differ between the groups (Table 3).

**Table 3:** Pulmonal parameters. Data shown as mean values and standard deviation

		VO	OAI	SA	SBA
PEEP	BLH	4 ± 0	4 ± 0	4 ± 0	4 ± 0
(cm H_2_O)	0 h	4 ± 0	6 ± 2	5 ± 2	5 ± 2
	6 h	4 ± 0	9 ± 2*	10 ± 2*	10 ± 2*
	12 h	4 ± 0	7 ± 3*	8 ± 1*	9 ± 2*
	18 h	4 ± 0	7 ± 3	7 ± 2	9 ± 3*
P_peak_	BLH	15 ± 2	16 ± 3	16 ± 2	15 ± 1
(cm H_2_O)	0 h	14 ± 1	28 ± 6*	25 ± 5*	25 ± 4*
	6 h	15 ± 2	28 ± 3*	28 ± 4*	27 ± 3*
	12 h	16 ± 2	28 ± 6*	27 ± 4*	26 ± 2*
	18 h	17 ± 2	30 ± 5*	26 ± 5*	25 ± 5*
V_T_	BLH	6 ± 0	6 ± 1	6 ± 0	6 ± 0
(ml/kg)	0 h	6 ± 0	7 ± 1	6 ± 0	6 ± 0
	6 h	6 ± 0	7 ± 1	6 ± 0	6 ± 0
	12 h	6 ± 0	6 ± 0	6 ± 0	6 ± 0
	18 h	6 ± 0	6 ± 0	6 ± 0	6 ± 0
etCO_2_	BLH	39 ± 3	38 ± 4	42 ± 2	42 ± 1
(mmHg)	0 h	36 ± 2	37 ± 4	39 ± 3	38 ± 2
	6 h	37 ± 4	39 ± 3	41 ± 2	41 ± 3
	12 h	37 ± 2	39 ± 3	39 ± 2	40 ± 3
	18 h	36 ± 2	39 ± 4	38 ± 2	40 ± 4
FiO_2_	BLH	40 ± 0	40 ± 0	40 ± 0	40 ± 0
(%)	0 h	100 ± 0	100 ± 0	100 ± 0	100 ± 0
	6 h	40 ± 0	55 ± 10*	59 ± 9*	65 ± 9*
	12 h	39 ± 4	48 ± 10	51 ± 13	59 ± 12*
	18 h	40 ± 0	45 ± 10	41 ± 3	54 ± 17
EVLWI	BLH	11 ± 1	10 ± 2	13 ± 3	14 ± 3
(ml/kg)	0 h	12 ± 1	19 ± 4*	20 ± 4*	26 ± 8*^#±^
	6 h	12 ± 2	21 ± 7*	21 ± 5*	25 ± 3*
	12 h	14 ± 2	17 ± 6	21 ± 6*	24 ± 3*^#^
	18 h	14 ± 3	17 ± 5	19 ± 4	23 ± 5*^#±^
paO_2_/FiO_2_	BLH	503 ± 65	496 ± 58	467 ± 63	449 ± 91
(mmHg)	0 h	544 ± 66	101 ± 28*	86 ± 34*	72 ± 20*
	6 h	452 ± 55	188 ± 70*	166 ± 40*	159 ± 53*
	12 h	453 ± 73	221 ± 48*	158 ± 45*	153 ± 53*
	18 h	400 ± 59	216 ± 50*	205 ± 43*	207 ± 105*
Wet-to-dry		5 ± 0	6 ± 1	6 ± 1	6 ± 1
Ratio					

^*^p < 0.05 vs VO; # p < 0.05 vs OAI; + p < 0.05 vs SA

Abbreviations: blh, baseline; etCO_2_, end tidal CO_2;_ EVLWI, extravascular lung water index; FiO_2_, inspiratory fraction of O_2_; OAI, lung injury by central venous injection of oleic acid; PEEP, positive end expiratory pressure; P_peak_, peak pressure; SA, statin treatment after lung injury; SBA, statin treatment 12 h before and directly after lung injury; VO, ventilation only; V_t_, tidal volume

Induction of lung injury led to a significant increase in heart rate and mean pulmonary arterial pressure in the OAI animals, the SA animals and the SBA animals, compared to the VO animals at all measured time points. Central venous pressure and cardiac index showed differences at some time points. No difference was found in mean arterial pressure, norepinephrine dose, and pulmonary capillary wedge pressure between the groups (Table 4).

**Table 4:** Cardiovascular parameters. Data shown as mean values and standard deviation

		VO	OAI	SA	SBA
HR	BLH	77 ± 10	77 ± 15	82 ± 9	82 ± 12
(min^−1^)	0 h	72 ± 9	124 ± 32*	127 ± 26*	112 ± 17*
	6 h	76 ± 15	119 ± 40*	131 ± 30*	118 ± 26*
	12 h	75 ± 18	117 ± 39*	126 ± 32*	112 ± 24*
	18 h	68 ± 8	112 ± 44*	142 ± 36*	115 ± 36*
MAP	BLH	72 ± 5	75 ± 11	74 ± 10	74 ± 11
(mmHg)	0 h	76 ± 8	73 ± 6	74 ± 8	74 ± 10
	6 h	74 ± 8	67 ± 8	65 ± 7*	64 ± 4*
	12 h	71 ± 12	69 ± 6	67 ± 10	63 ± 5
	18 h	68 ± 8	67 ± 7	67 ± 9	60 ± 4
MPAP	BLH	13 ± 4	16 ± 3	15 ± 2	16 ± 4
(mmHg)	0 h	12 ± 3	38 ± 4*	35 ± 4*	38 ± 4*
	6 h	14 ± 4	30 ± 5*	30 ± 4*	31 ± 4*
	12 h	16 ± 2	27 ± 4*	28 ± 5*	31 ± 6*
	18 h	15 ± 2	27 ± 2*	27 ± 4*	29 ± 7*
CVP	BLH	6 ± 2	6 ± 3	5 ± 3	4 ± 2
(mmHg)	0 h	6 ± 3	7 ± 4	6 ± 4	6 ± 3
	6 h	8 ± 4	8 ± 3	7 ± 2	8 ± 2
	12 h	6 ± 2	9 ± 3	8 ± 2	9 ± 2
	18 h	6 ± 1	10 ± 3*	7 ± 2^#^	10 ± 2*^±^
PCWP	BLH	7 ± 1	8 ± 1	7 ± 3	6 ± 1
(mmHg)	0 h	7 ± 2	9 ± 2	8 ± 4	8 ± 2
	6 h	7 ± 1	8 ± 2	8 ± 3	8 ± 2
	12 h	7 ± 2	9 ± 2	8 ± 2	8 ± 2
	18 h	7 ± 2	10 ± 1	8 ± 3	9 ± 1
CI	BLH	3.41 ± 0.4	3.34 ± 0.9	3.65 ± 0.6	3.37 ± 0.7
(l/min/m^2^)	0 h	3.26 ± 0.4	3.87 ± 0.7	4.04 ± 0.8	3.47 ± 0.9
	6 h	3.40 ± 0.7	3.87 ± 0.9	4.14 ± 1.0	3.53 ± 0.9
	12 h	3.61 ± 0.6	4.44 ± 1.4	4.64 ± 1.2	4.19 ± 0.7
	18 h	3.28 ± 0.3	4.83 ± 1.4*	5.49 ± 1.5*	5.02 ± 1.4*
Norepinephrine	BLH	0.04 ± 0.1	0.00 ± 0.0	0.01 ± 0.0	0.00 ± 0.0
(μg/kg/min)	0 h	0.00 ± 0.0	0.55 ± 0.6	0.49 ± 0.7	0.31 ± 0.3
	6 h	0.01 ± 0.0	0.58 ± 1.1	0.57 ± 0.6	0.41 ± 0.3
	12 h	0.03 ± 0.0	0.66 ± 1.0	1.02 ± 0.8	1.05 ± 1.3
	18 h	0.01 ± 0.0	1.09 ± 1.4	1.15 ± 1.0	1.66 ± 1.9

^*^p < 0.05 vs VO; # p < 0.05 vs OAI; + p < 0.05 vs SA

Abbreviations: blh, baseline; CI, cardiac index; CVP, central venous pressure; HR, heart rate; MAP, mean arterial pressure; MPAP, mean pulmonary arterial pressure; OAI, lung injury by central venous injection of oleic acid; PCWP, pulmonary capillary wedge pressure; SA, statin treatment after lung injury; SBA, statin treatment 12 h before and directly after lung injury; VO, ventilation only

### Lungs and blood

**Fig. 1:**
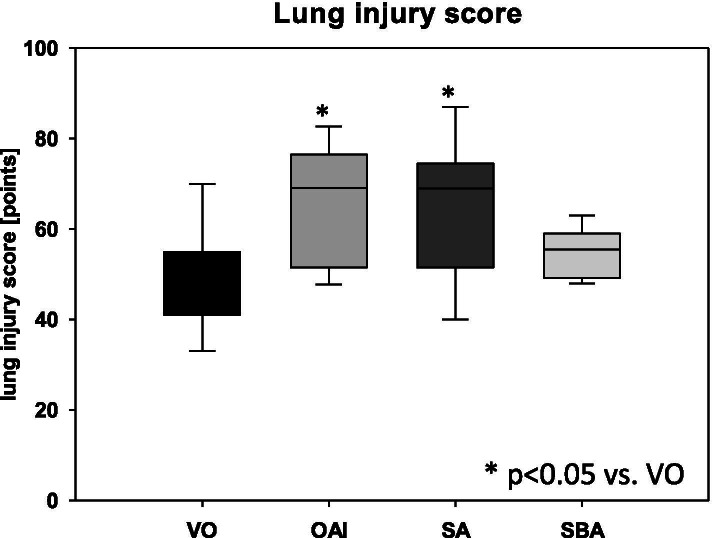
Lung injury score in the treated animals as measured by histopathology in HE-stained lungs. Data are mean with standard deviation presented as box plots. 8 animals per group were used

**Fig. 2:**
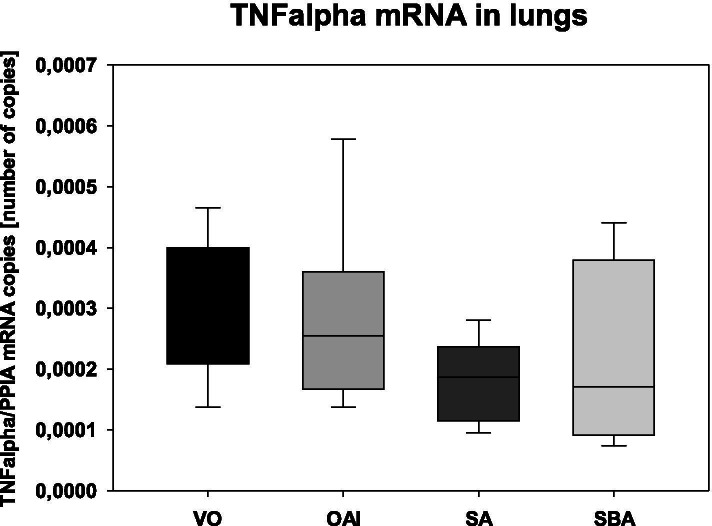
TNFalpha mRNA concentration in the lungs as measured by RT-PCR. Data are mean with standard deviation presented as box plots. 8 animals per group were used

The animals in the OAI group showed a significantly higher lung injury score compared to ventilation-only animals (p < 0.05; Fig. 1). The animals of the SA group had a higher lung injury score than the animals of the VO group (p < 0.05; Fig. 1). There was no significant difference in the lung injury scores between the SBA animals and the ventilation-only animals (p = 0.43; Fig. 1). There was no difference between OAI and SA (p = 0.81; Fig. 1), between OAI and SBA (p = 0.15; Fig. 1), or between SA and SBA (p = 0.10; Fig. 1). Expression of TNFalpha mRNA showed no difference between the groups 18 h after induction of lung injury (p = 0.25; Fig. 2). No difference was found in the wet-to-dry ratio between the groups (p = 0.10; Table 3).

**Fig. 3:**
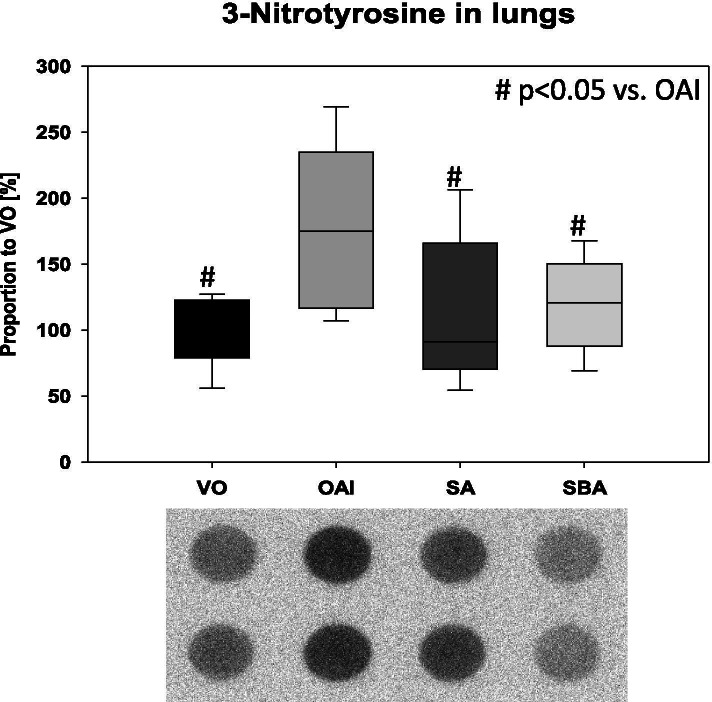
3-Nitrotyrosine-positive protein content in the lungs of treated animals as measured by dot blot analysis using a specific 3-nitrotyrosine antibody. Data are mean with deviation presented as box plots. 8 animals per group were used. 2 representative dot blots per group are shown

We found a significantly higher concentration of 3-nitrotyrosine in the lungs of the OAI animals compared to VO, SBA, or SA (p < 0.05 in all, Fig. 3). There was no difference between the other groups (VO vs. SBA p = 0.54, VO vs. SA p = 0.6, SBA vs. SA p = 0.59; Fig. 3).

**Fig. 4:**
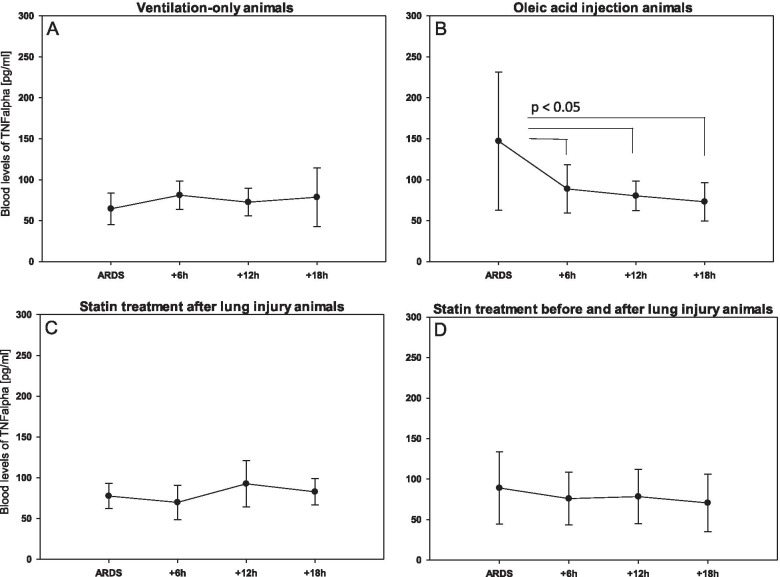
Time course of blood levels of TNFalpha as measured by ELISA. Data are mean with standard deviation of 8 animals per group. **A** Ventilation-only. **B** Oleic acid injection. **C** Statin treatment after lung injury. **D** Statin treatment before and after lung injury

There was no change in blood serum concentrations of TNFalpha over time in animals in the VO (p = 0.56; Fig. 4), SBA (p = 0.29; Fig. 4), or SA (p = 0.20; Fig. 4) groups. In the animals in the OAI group, TNFalpha was increased directly after induction of lung injury (p < 0.05; Fig. 4) but decreased afterwards to values similar to animals of the other groups.

There was no significant difference in TNFalpha serum concentrations between the groups directly after ARDS induction (p = 0.06), 6 h later (p = 0.53), 12 h later (p = 0.48), or 18 h later (p = 0.83).

### Brain

**Fig. 5:**
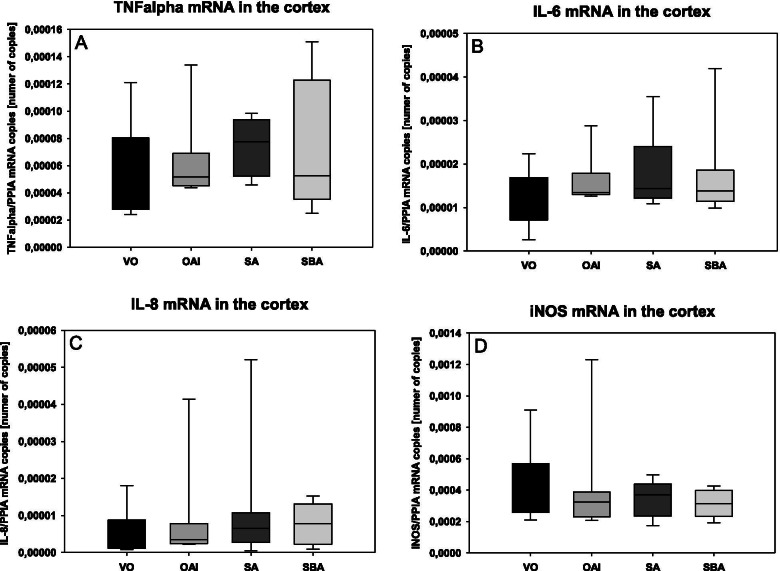
mRNA levels of different cytokines in the cortices of treated animals as measured by RT-PCR. Data are mean with standard deviation presented as box plots. 8 animals per group were used. **A** TNFalpha. **B** IL-6. **C** IL-8. **D** iNOS

The expression of TNFalpha mRNA in the cortex of the animals was not different between the groups (Fig. 5), nor were any differences observed in the expression of IL-6 mRNA (p = 0.35), IL-8 mRNA (p = 0.68), or iNOS mRNA (p = 0.74) in the cortex of the animals between the groups (Fig. 5).

**Fig. 6:**
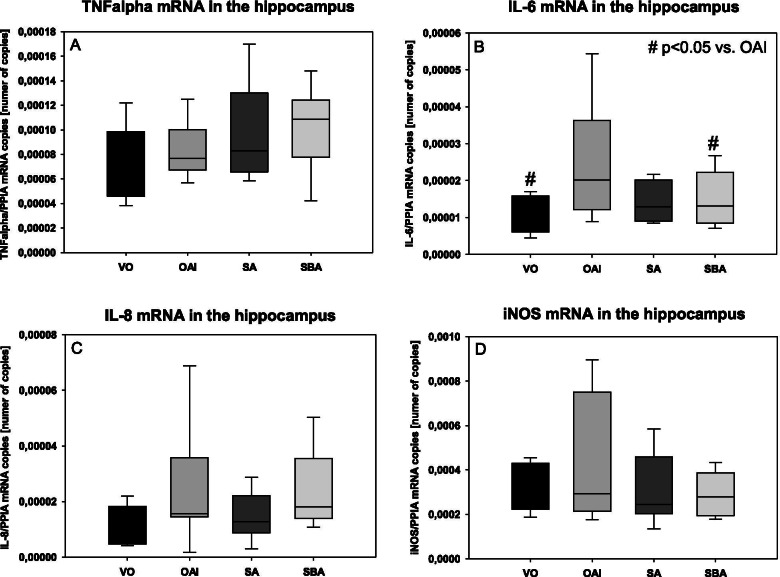
mRNA levels of different cytokines in the hippocampus of treated animals as measured by RT-PCR. Data are mean with standard deviation presented as box plots. 8 animals per group were used. **A** TNFalpha. **B** IL-6. **C** IL-8. **D** iNOS

In the hippocampus of the animals, there was no difference in the levels of TNFalpha, IL-8, or iNOS mRNA between the groups (Fig. 6). IL-6 mRNA expression was significantly increased in the hippocampus of the OAI animals compared to the VO animals (p < 0.05; Fig. 6). In the hippocampus of animals of the SBA group, IL-6 mRNA expression was significantly lower than in the OAI group (p < 0.05; Fig. 6). The animals in the SA group showed a tendency for lower IL-6 mRNA expression in their hippocampus compared to the OAI animals, but lacked significance (p = 0.08; Fig. 6). There was no difference in IL-6 mRNA concentration in the hippocampus of VO compared to SBA (p = 0.75; Fig. 6), VO compared to SA (p = 0.55; Fig. 6), or SBA compared to SA (p = 0.91; Fig. 6).

**Fig. 7:**
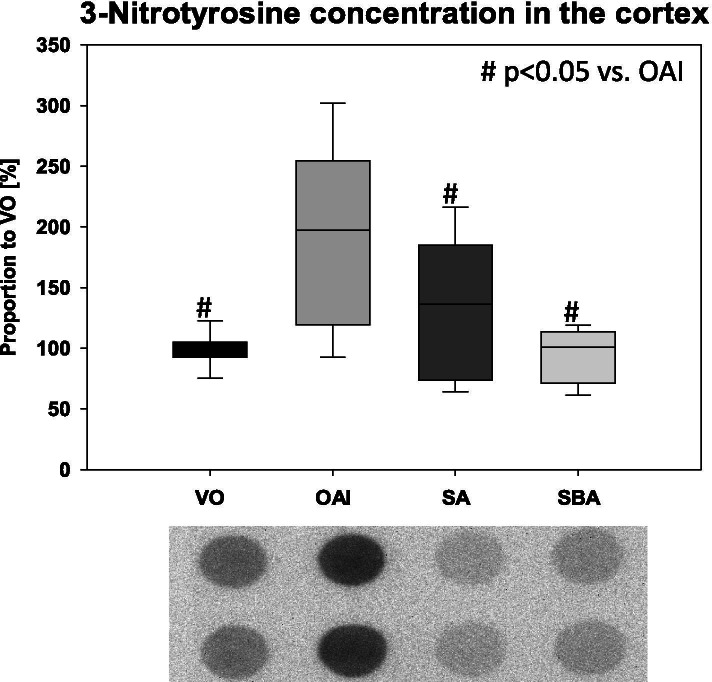
3-Nitrotyrosine-positive protein content in the cortices of treated animals as measured by dot blot analysis using a specific 3-nitrotyrosine antibody. Data are mean with standard deviation presented as box plots. 8 animals per group were used. 2 representative dot blots per group are shown

Notably, the levels of 3-nitrotyrosine-positive proteins were significantly increased in the cortex of the animals of the OAI group compared to animals in the VO (p < 0.001), SBA (p < 0.001), and SA (p < 0.05) groups (Fig. 7).

## Discussion

In the present study, we found evidence of cerebral cytokine production and nitro-oxidative stress within the first day of inducing lung injury. Rosuvastatin treatment reduced IL-6 mRNA production and 3-nitrotyrosine concentrations in the brains of the animals.

Central venous injection of oleic acid is an established animal model of acute lung injury [35], characterised by alveolar haemorrhage, intravascular thrombosis, PMN infiltration, and an increase in pulmonary microvascular permeability. These conditions lead to severe ventilation/perfusion (V/Q) mismatch and increased shunt, which results in hypoxaemia and increased mean airway pressures. Furthermore, pulmonary oedema, characterised by elevation of extravascular lung water and leakage of protein-rich fluid into the airspace and interstitium, develops. These hemodynamic effects lead to myocardial depression, early systemic hypotension, and pulmonary hypertension [2]. Our study animals reliably developed the described pathologic changes. Some of these pathologies ameliorated (paO_2_, MPAP) over time. This is in contrast to the study by Matute-Bello et al., who reported the maximal effect of oleic acid after 12 h [2]. This may be attributable to persistent protective ventilation, which may prevent the aggravation of inflammation through mechanical ventilation by minimizing alveolar stress, strain, and cyclic recruitment and derecruitment [36]. Even though treatment with rosuvastatin reduced the pulmonary damage assessed by histopathology, it did not improve oxygenation. This could explain partly the results of a clinical trial that showed no improvement of “hard” outcome parameters, such as mortality or ventilator-free days, in ARDS patients treated with statins [18].

TNFalpha is a central cytokine in the inflammatory response. It is involved in the development of various pulmonary diseases, including acute lung injury and ARDS [37]. It also plays an important role in the cognitive decline of patients with Alzheimer’s disease [38] and is associated with postoperative delirium and cognitive dysfunction [39]. The lungs of our animals showed no intergroup differences in TNFalpha concentration. TNFalpha accumulates usually very rapidly after an insult to the lungs [40]; thus, expect higher levels were expected, at least in the animals of the oleic acid group. As mentioned above, the absence of TNFalpha may be due to the protective ventilation in our study setting, which might have prevented further biotrauma to the lungs and thereby enabled the amelioration of lung function and immune response. Nonetheless, oleic acid-induced lung injury led to persistent damage to the lungs, as shown by a significant increase in the lung injury score in the animals of the oleic acid group compared to ventilation-only animals. The lungs of the animals in the SA group showed a similar result, whereas the damage to the lungs of the SBA animals was non-significantly lower than that of the oleic-acid animals, but simultaneously non-significantly higher than that of the VO-animals. Dot blot revealed significantly higher 3-nitrotyrosine concentrations in the animals of the oleic acid groups compared to all other groups, whereas there was no difference between the animals in the VO, SA, or SBA group. This could be attributable to the antioxidant capacity of rosuvastatin.

The time course of TNFalpha blood levels supports the idea of an only short-term immune response in our study setting. Immediately after induction of lung injury, TNFalpha increased significantly in the blood samples of the animals in the OAI group but decreased rapidly after and reached baseline values. The comparison of TNAalpha blood levels in OAI animals with VO animals directly after ARDS induction missed significance, but showed a strong tendency to higher values in OAI animals (OAI 147 ± 85 pg/ml, VO 65 ± 19 pg/ml p = 0.06). This is similar to the study of Bickenbach et al., who found a non-significant tendency for an increase in serum TNFalpha in an animal model of acid aspiration-induced lung injury [12]. Statin treatment blunted this initial increase, which may be due to the anti-inflammatory properties of rosuvastatin.

Circulating TNFalpha can compromise the blood brain barrier [41]. Furthermore, it can activate microglia cells through receptors present in cerebral blood vessels [42]. Activated microglia cells, in turn, produce and release TNFalpha [43]. In our study, we found no difference in cerebral or hippocampal TNFalpha mRNA concentrations between the groups. Whether this is indicative of a non-activation of microglia cells or of a transient effect parallel to the blood levels of TNFalpha remains unclear. IL-8, another cytokine, showed no difference between the groups either. A third indicator of cerebral inflammation is IL-6. In our study, IL-6 mRNA significantly increased in the hippocampus of the OA animals compared to the VO animals. Bellaver et al. examined cerebral cytokine production in animals treated with intraperitoneal lipopolysaccharide (LPS) and found an increase in hippocampal IL-6 production [44]. Intraperitoneal application of LPS is a sepsis model, not a pure ARDS model, as sepsis leads to a fast breakdown of the blood–brain barrier, causing cerebral inflammation [45]. Cytokine release in ARDS is lower than in sepsis; therefore, the breakdown—or potential breakdown—of the blood–brain barrier may take longer, which could explain these results. Treatment with rosuvastatin seems to reduce the hippocampal production of IL-6.

Nitric oxide (NO) production by iNOS is thought to cause neuronal damage under several conditions [46], especially in early inflammatory syndromes [47]. NO production in the presence of superoxide leads to peroxynitrite formation, which in turn leads to the nitration of various molecules and subsequently to neuronal cell death [48]. We found no difference in iNOS mRNA between the groups in cortical nor in hippocampal tissue. Nevertheless, we found an increase in 3-nitrotyrosine production. This effect was significantly blunted in the animals of the statin groups, perhaps due to its antioxidant properties. This is concordant with a study by Kadoi et al., who found an increase in cerebral nitrotyrosine formation 24 h after caecal ligation and puncture-induced sepsis [49]. The missing induction of iNOS mRNA may be due to the time course used in our study. In mice, hippocampal iNOS increased significantly 24 h after LPS-induced sepsis [50], whereas there was no increase in iNOS in other brain regions.

Our study features some limitations. Lung injury induced by injection of oleic acid is a well-established model for acute ARDS and is characterized by a profound change in oxygenation due to microvascular thrombosis, PMN infiltration, necrosis, and leakage of protein-rich fluid into the airspace, with extravascular lung water accumulation [51]. All these features were reproduced in our study after the onset of ARDS but improved during the course of the study. In particular, the wet-to-dry ratio as a marker for pulmonary oedema did not differ between the lung injury group and the control group. Even though the pulmonary damage score was significantly higher in the lung injury group, the lack of difference in the wet-to-dry ratio indicates a certain amelioration of the lung injury, possibly due to the lung-protective ventilator setting. Furthermore, the observed amelioration could go along with an amelioration of lung inflammation. This amelioration, in turn, could lead to an attenuated cytokine release in the blood with a less pronounced systemic inflammatory response and accordingly reduced cerebral inflammation compared to septic conditions. Another limitation is the duration of our experiment. Usually, patients with ARDS are in need of mechanical ventilation for several days [52], whereas our experiment was limited to 18 h. This may be too short to induce a cerebral reaction not mediated by the nervous system but by the circulatory system. The systemic immune response usually lags some days after localised infection. Lastly, we examined only the mRNA of the cerebral cytokines, not proteins. We did this to rule out an overspill of plasmatic cytokines into the brain. Finally, oleic acid is known to transiently open the blood–brain-barrier [53, 54], so maybe the observed changes in cerebral IL-6 and nitro-tyrosine are due to an oleic acid effect. This seems rather unlikely, because to examine effects of oleic acid on the blood–brain-barrier, it has to be injected intra-arterial. It seems doubtful that a relevant amount of oleic acid reaches the brain after passing through the pulmonal vasculature.

## Conclusion

This is the first study to show an increase in cerebral nitro-oxidative stress after experimental ARDS in pigs and an attenuation of this effect through treatment with rosuvastatin. Previous studies failed to show an effect of statin treatment on “hard” outcome parameters, such as mortality, length of hospital stay, or ventilator-free days in patients with sepsis-associated ARDS [19, 55]. Some studies have found a significant increase in quality of life and cost reduction in patients treated with simvastatin compared to a placebo [22]. Considering these with our results, it can be concluded that treating ARDS patients with statins may not affect the immediate outcome but could have positive effects in the long run. These effects may be due to the reduction of cerebral inflammation and accompanying neurocognitive disorders. Further investigations regarding possible cerebral inflammation after a longer duration of ARDS and a possible protective effect of statin treatment are necessary.

## Supplementary Information

**Additional file 1**

## Data Availability

The datasets generated and analysed during the current study are included within the article and its additional file (see additional file 1).

## Abbreviations

ARDS: Acute respiratory distress syndrome
EVLWI: Extravascular lung water index
FiO_2_: Fraction of inspired oxygen
OAI: Lung injury by central venous injection of oleic acid
PEEP: Positive end-expiratory pressure
PiCCO: Pulse contour cardiac output catheter
P_peak_: Peak pressure SA statin treatment
SA: Statin treatment after lung injury
SBA: Statin treatment 12 h before and directly after lung injury
VO: Ventilation only
Vt: Tidal volume
