# Supplementary material for: Influence of rosuvastatin treatment on cerebral inflammation and nitro-oxidative stress in experimental lung injury in pigs
Source: BMC Anesthesiol. 2021 Sep 13;21:224. doi: 10.1186/s12871-021-01436-0 (PMC8435760; doi:10.1186/s12871-021-01436-0)
Supplement: Supplementary file 1 — Additional file 1 [file 12871_2021_1436_MOESM1_ESM.pdf]

Influence of rosuvastatin treatment on cerebral inflammation and nitro-oxidative stress in experimental lung injury in pigs – Additional file 1

Jens Kamuf[1*], Andreas Garcia Bardon[1], Alexander Ziebart[1], Robert Rümmler[1], Johannes Schwab[1], Mobin Dib[2], Andreas Daiber[2], Serge C Thal[1], Erik K Hartmann[1]

Datasets generated during this study

mRNA Datasets [number of mRNA copies]

TNFalpha Cortex

VO	OAI	SA	SBA
6,2800e-5	4,3900e-5	9,8500e-5	1,5100e-4
3,1300e-5	5,7300e-5	4,5900e-5	4,1800e-5
8,2000e-5	4,3800e-5	9,1800e-5	4,6200e-5
1,2100e-4	1,3400e-4	5,1500e-5	3,3200e-5
2,4200e-5	4,9300e-5	9,4300e-5	1,1900e-4
7,4800e-5	7,2800e-5	8,8300e-5	5,9200e-5
2,6600e-5	5,0300e-5	5,4600e-5	2,4800e-5
7,6100e-5	5,3100e-5	6,6800e-5	1,2400e-4

TNFalpha Hippocampus

VO	OAI	SA	SBA
6,0100e-5	7,7100e-5	8,7500e-5	1,2200e-4
4,1200e-5	1,2500e-4	6,4700e-5	7,4300e-5
1,2200e-4	8,5900e-5	1,7000e-4	1,1500e-4
8,4000e-5	1,0500e-4	6,9000e-5	1,2500e-4
8,4700e-5	7,6600e-5	9,7200e-5	1,4800e-4
1,0100e-4	6,6300e-5	5,8300e-5	4,2300e-5
9,1300e-5	5,6900e-5	7,8500e-5	8,7200e-5
3,8300e-5	6,9800e-5	1,4100e-4	1,0200e-4

IL-6 Cortex

VO	OAI	SA	SBA
2,2400e-5	1,3000e-5	2,4500e-5	1,6400e-5
1,7300e-5	1,3600e-5	1,2100e-5	1,4100e-5
9,9000e-6	1,9200e-5	3,5500e-5	4,2000e-5
9,3800e-6	2,8800e-5	1,0900e-5	9,9000e-6
1,5600e-5	1,3000e-5	2,2700e-5	1,9300e-5
2,6400e-6	1,3400e-5	1,6300e-5	1,3600e-5
6,9100e-6	1,3800e-5	1,2200e-5	1,1100e-5
7,8000e-6	1,2600e-5	1,2400e-5	1,2400e-5

IL-6 Hippocampus

VO	OAI	SA	SBA
1,3600e-5	5,4400e-5	1,9500e-5	1,1900e-5
1,6000e-5	2,1400e-5	2,1700e-5	2,6800e-5
1,5300e-5	4,0200e-5	9,3600e-6	2,4500e-5
1,7000e-5	1,9000e-5	8,4300e-6	7,6900e-6
8,1500e-6	2,4700e-5	1,5000e-5	1,0700e-5
1,1500e-5	8,8800e-6	8,8300e-6	1,4300e-5
4,5200e-6	9,8800e-6	1,0800e-5	7,0700e-6
5,3700e-6	1,8900e-5	2,0400e-5	1,5400e-5

IL-8 Cortex

VO	OAI	SA	SBA
1,8100e-5	7,8400e-6	6,5100e-6	8,6300e-6
1,2800e-6	3,4400e-6	5,2100e-7	1,5300e-5
1,0200e-5	4,1400e-5	5,7600e-6	1,7100e-6
4,3200e-6	3,5000e-6	1,0700e-5	8,6600e-6
1,1000e-6	2,4500e-6	5,2100e-5	1,4600e-5
3,2600e-6	3,0500e-6	9,0800e-6	8,7800e-7
2,2400e-6	2,2200e-6	2,7200e-6	3,6700e-6
7,5900e-7			6,9400e-6

IL-8 Hippocampus

VO	OAI	SA	SBA
2,2000e-5	6,8900e-5	7,7100e-6	5,0300e-5
7,7900e-6	1,5100e-5	1,2300e-5	1,3400e-5
1,9500e-5	1,6800e-5	2,8700e-5	1,5200e-5
1,4400e-5	1,4300e-5	3,0400e-6	1,0700e-5
5,1500e-6	1,7100e-6	1,2000e-5	2,0500e-5
8,2300e-6	1,6300e-5	1,3300e-5	2,0200e-5
4,6200e-6	1,4900e-5	2,3300e-5	4,0500e-5
4,1200e-6	4,2200e-5	1,8800e-5	1,6100e-5

iNOS Cortex

VO	OAI	SA	SBA
9,1000e-4	2,0800e-4	4,4800e-4	2,2000e-4
6,0800e-4	2,1800e-4	3,7400e-4	3,7600e-4
2,5600e-4	1,2300e-3	1,7500e-4	2,7100e-4
2,1000e-4	4,0300e-4	4,9600e-4	4,0500e-4
2,6900e-4	2,7000e-4	4,1100e-4	3,0600e-4
3,4700e-4	3,4600e-4	3,6700e-4	4,2600e-4
2,6000e-4	3,2800e-4	2,2100e-4	1,9200e-4
4,5400e-4	3,1800e-4	2,7600e-4	3,2300e-4

iNOS Hippocampus

VO	OAI	SA	SBA
4,3300e-4	8,7300e-4	2,5100e-4	1,7900e-4
4,5600e-4	3,5800e-4	5,8400e-4	2,4300e-4
2,8200e-4	8,9500e-4	1,3400e-4	2,7300e-4
1,8700e-4	2,0900e-4	5,0500e-4	3,7600e-4
4,1900e-4	1,7600e-4	2,3700e-4	2,8500e-4
4,0800e-4	2,2900e-4	2,2100e-4	4,3400e-4
2,5900e-4	3,8500e-4	3,2000e-4	1,7700e-4
2,1100e-4	2,2600e-4	1,9600e-4	3,9100e-4

TNFalpha lungs

VO	OAI	SA	SBA
4,6500e-4	2,4700e-4	2,3400e-4	4,0600e-4
3,6200e-4	3,0300e-4	9,5800e-5	9,0100e-5
2,5900e-4	1,3700e-4	1,7400e-4	2,3100e-4
1,3700e-4	2,5900e-4	1,0200e-4	1,1100e-4
4,0000e-4	2,5000e-4	2,3800e-4	2,9700e-4
2,0900e-4	1,4000e-4	1,5400e-4	9,5500e-5
2,2700e-4	3,7900e-4	2,8000e-4	4,4100e-4
	5,7800e-4	1,9900e-4	7,3600e-5

ELISA Datasets [pg/ml]

VO

ARDS	+6h	+12h	+ 18h
63,0020	85,6360		155,1780
53,8450	61,4670	99,3860	84,6240
75,2700	81,0190	50,6120	54,9600
63,1620	99,1550	60,2940	67,7980
	64,4020	65,3320	78,7450
48,5220	68,7520	69,2580	43,3710
45,6990	111,5360	89,3990	95,3870
102,0070	76,7750	73,2390	49,0610

OAI

ARDS	+6h	+12h	+ 18h
97,2760	90,5080	102,8960	105,6950
183,6830	63,3120	74,4190	67,1650
194,4720	69,0700	61,1850	39,7510
281,2080	70,2830	98,5960	73,2500
67,1300	62,3260	60,5540	61,0830
60,2050	103,8890	61,6360	67,7150
68,9060	100,4280	86,6220	60,7740
223,9440	149,6630	96,2320	108,8150

SA

ARDS	+6h	+12h	+ 18h
	113,1800	153,8640	81,3820
84,5210	56,7440	64,8910	63,9310
78,3490	75,8330	72,9440	76,9120
59,8730	76,8330	80,5470	72,9090
107,7160	64,0030	86,0850	106,4430
74,7540	47,8030	85,6020	65,1760
68,6660	74,8180	111,5680	100,6860
69,5200	48,9960	85,3990	94,7880

SBA

ARDS	+6h	+12h	+ 18h
84,3890	40,7290	47,3230	57,9020
84,5985	125,5940	95,0920	61,0890
67,4470	62,0100	51,0110	49,9280
177,3540	119,5690	142,2720	155,3160
280,1130	59,8020	85,0370	41,3950
	94,3430	98,7760	64,5740
65,0320	54,9780	47,1540	67,6060
55,3950	50,4350	60,4390	67,0900

Dot Blot Datasets [% VO]

Cortex

VO	OAI	SA	SBA
82,9496	262,5702		101,0095
72,2628	313,2507	220,1360	119,1248
114,7859	191,4556	150,3907	111,7087
126,1862	223,9539	160,1761	118,3298
102,7970	230,5122	62,2777	100,6858
101,0185	274,8437	78,5981	83,8165
97,5759	195,3059	81,6371	66,8987
104,0035	199,0329	73,5371	59,8484
98,4205	192,2659	72,2738	64,7688
103,9286	91,9277	184,8465	114,0875
91,0209	95,3685	136,2894	112,9165
105,0505	94,0839	200,9764	86,8637

Lungs

VO	OAI	SA	SBA
62,0990	115,4934		110,7158
82,3979	130,1288	146,3013	81,6547
108,8358	207,9933	84,8042	69,1662
124,7147	243,2694	65,0839	54,0564
97,8722	255,8269	93,9197	73,5887
124,0804	274,9097	87,7975	55,5683
112,3380	110,6192	120,7519	209,8760
109,8674	118,9090	131,8919	198,0349
77,7947	105,3625	116,2316	184,1823
53,3861	152,5051	163,1758	102,2121
118,4230	209,2435	150,2181	92,3942
128,1909	197,1244	169,0041	90,2576

Lung injury score dataset [score]

VO	OAI	SA	SBA
39,7500	62,0000	56,0000	87,0000
70,0000	67,2500	48,0000	67,0000
53,5000	82,7500	60,0000	51,0000
33,0000	72,0000	55,0000	73,0000
55,0000	78,0000	63,0000	53,0000
55,0000	71,0000	50,0000	75,0000
45,0000	48,0000	49,0000	71,0000
48,0000	47,7500	56,0000	40,0000
